# A Fungistatic Strategy Using a Shear-Thinning pH-Responsive CMCS-OHA-Lp/Lr Hydrogel for Vulvovaginal Candidiasis

**DOI:** 10.3390/pharmaceutics17040527

**Published:** 2025-04-17

**Authors:** Yuanmin Zhao, Xiu Yang, Jiale Han, Chaoqi Huang, Mengliu Shao, Yan Yang, Qingliang Yang, Gensheng Yang

**Affiliations:** College of Pharmaceutical Science, Zhejiang University of Technology, Hangzhou 310014, China; m17861405730@163.com (Y.Z.); 221123070155@zjut.edu.cn (X.Y.); 221123070231@zjut.edu.cn (J.H.); 211123070137@zjut.edu.cn (C.H.); 211123070059@zjut.edu.cn (M.S.); yangyan10@zjut.edu.cn (Y.Y.)

**Keywords:** shear-thinning vaginal hydrogel, probiotic gel, vulvovaginal candidiasis, Lactobacillus, in situ release

## Abstract

**Background**: Vulvar vaginal candidiasis (VVC) is a type of vaginitis resulting from a Candida infection of the vaginal mucosa. Traditional treatments using antibiotics often lead to resistance and disrupt the vaginal microenvironment, causing ongoing problems for patients. In response to these challenges, this study introduces a multifunctional intelligent responsive probiotic hydrogel designed to modulate the vaginal microecological environment to combat *Candida albicans* infection. **Methods**: The innovative CMCS-OHA-Lp/Lr hydrogel was formulated using oxidized hyaluronic acid (OHA) and carboxymethyl chitosan (CMCS) as carriers, incorporating *Lactobacillus plantarum* (Lp) and *Lactobacillus rhamnosus* (Lr) as active components. Comprehensive characterization of the CMCS-OHA-Lp/Lr hydrogel revealed its chemical structure, rheological properties, rapid self-healing properties, gel degradation, and the release of lactobacilli in vitro. **Results**: The findings demonstrated that the hydrogel’s cross-linking conferred significant physical properties. In addition, the in vitro release study of Lactobacillus showed that the cumulative release rates of Lp and Lr in the medium with pH 5.5 were 83.50 ± 2.70% and 73.31 ± 2.22%, which proved the pH-responsive release characteristics of probiotics in acidic vaginal environments. Furthermore, the storage activity of Lactobacillus indicated that the survival rates of the CMCS-OHA-Lp and CMCS-OHA-Lr hydrogels were 86.90 ± 0.20% and 85.50 ± 0.56%, respectively, proving that encapsulation within the hydrogels significantly enhanced the storage stability of probiotics. In vivo studies further confirmed that the hydrogel alleviated vulval edema symptoms and reduced *C. albicans* colonies in the vagina, thereby mitigating vaginal inflammation. **Conclusions**: In conclusion, this pH-responsive, self-healing, and shear-thinning hydrogel offers a promising approach for the clinical treatment of VVC and serves as an effective probiotic delivery vehicle.

## 1. Introduction

Vulvovaginal candidiasis (VVC), commonly referred to as *C. albicans* vaginitis, is a fungal infection of the vagina caused by the Candida pathogen [1,2,3]. VVC is the second most prevalent gynecological inflammation after bacterial vaginitis, VVC affects the health and well-being of millions of women annually, thereby constituting a significant public health concern [4,5]. In severe instances, VVC can progress to cervicitis and other complications. Studies indicate that 75% of healthy women will experience VVC at least once during their reproductive years, with 40% to 45% experiencing two or more episodes, and 5% to 10% suffering from recurrent infections [6,7,8].

The traditional treatment for VVC predominantly involves the use of azole antifungal agents, such as fluconazole, miconazole, and ketoconazole [9,10]. Antifungals can be administered either orally or via local vaginal delivery methods [11], including suppositories, vaginal capsules, and gels. While these treatments are effective in rapidly eliminating harmful fungi and significantly reducing inflammation, they are not without drawbacks [12,13,14]. A variety of negative effects are linked to the oral administration of azole antifungals, including headaches, abdominal pain, nausea, and severe liver toxicity, which can escalate to liver damage or even liver failure [15]. In contrast, local vaginal administration minimizes systemic toxicity and reduces the risk of drug interactions. However, dosage forms such as vaginal suppositories and capsules can significantly increase patient discomfort, manifesting as itching, pain, and burning. Moreover, the use of topical antifungals may disrupt the protective vaginal flora, potentially leading to recurrent infections by disturbing the microecological balance [16]. In order to overcome the limitations of basic antifungal therapy and enhance the bioavailability of antibiotics, nanodrug delivery systems such as lipid nanoparticles and liposomes containing antifungal drugs have been reported for the treatment of VVC [17]. However, the extensive application of antifungals has contributed to a marked increase in resistance among *C. albicans*, resulting in the emergence of numerous drug-resistant and cross-resistant strains [18]. This resistance diminishes drug sensitivity and perpetuates a vicious cycle of treatment challenges [19]. In addition to traditional treatment methods, the research on new alternative treatment methods is also increasing in recent years, the most representative of which is photodynamic therapy [20,21]. The correct use of alternative therapies can effectively promote the treatment of VVC, but it is important to note that some chemical products may have adverse side effects.

To effectively address these issues, it is crucial to explore innovative therapeutic strategies [5,22], with one of the most promising approaches being to manipulate the vaginal microecology [23,24]. Under normal conditions, the balance of the vaginal microbial flora is predominantly maintained by lactic acid bacteria [25,26]. These microorganisms engage in a symbiotic relationship with their human hosts, offering essential nutrients and shelter, while simultaneously protecting the host from various harmful pathogens [27]. The vaginal flora of healthy women is dominated by Lactobacillus, according to extensive study of vaginitis and the vaginal microenvironment, defending against pathogens through multiple mechanisms [28,29]. Lactobacillus regulates the production of antimicrobial compounds, such as hydrogen peroxide, which is toxic to anaerobic microorganisms [30]. Additionally, Lactobacillus binds to the surfaces of vaginal epithelial cells, competitively preventing the attachment and infection of other microorganisms [31]. It also produces various antimicrobial peptides, including bacteriocins [26,32,33] and biosurfactants, and promotes autophagy, a process involving the phagocytosis and degradation of intracellular bacteria and viruses. Furthermore, a low vaginal pH is attributed to the production of lactic acid by Lactobacillus, creating an acidic microenvironment [16,34].

The vitality and stability of Lactobacillus are essential for maintaining its therapeutic effects as a probiotic for vaginal diseases [35,36]. However, Lactobacillus is extremely sensitive to changes in its environment, including pH, temperature, and storage time, which can significantly reduce its survival rates [37,38,39]. To addresses this challenge, it is crucial to select appropriate delivery carriers that enhance the viability and bioavailability of probiotics [40].

**Scheme 1:**
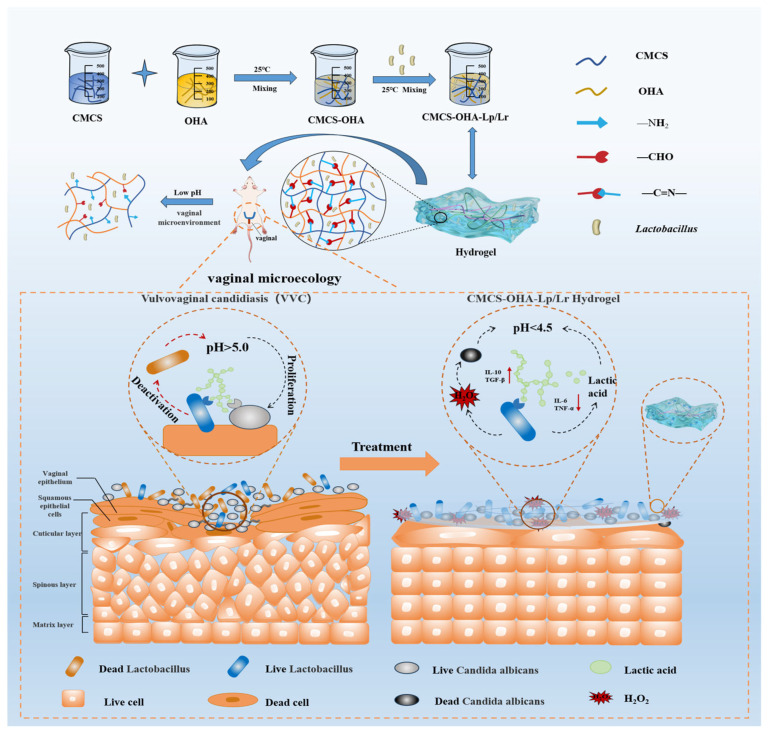
Preparation of multifunctional hydrogel and its mechanism in the treatment of VVC.

An effective carrier for probiotics like Lactobacillus must be capable of completely encapsulating probiotics to provide sufficient protection [41] while also being able to fully release them in situ to exert therapeutic effects [42,43]. Carboxymethyl chitosan (CMCS) is widely utilized as hydrogel-forming polymers in biomedical applications, owing to its excellent biocompatibility, tunable mechanical properties, and sustained permeability to oxygen and nutrients [44,45]. Oxidized hyaluronic acid (OHA) is produced through the oxidation of hyaluronic acid. The active aldehyde groups in OHA can self-assemble into hydrogels via a Schiff base reaction with amino groups. OHA retains the biocompatibility and biodegradability properties of hyaluronic acid while also exhibiting good cross-linking properties. This modification was particularly desirable for our study as it enabled rapid hydrogel formation under physiological conditions without requiring external energy input. To date, research on CMCS and OHA hydrogels has predominantly concentrated on their roles in wound healing [46] and antimicrobial therapies, while their potential as engineered platforms for probiotic encapsulation in vaginal drug delivery systems remains largely unexplored [47]. Herein, our study aims to design and develop a hydrogel delivery system for Lactobacillus species, utilizing CMCS and OHA [44]. This system seeks to achieve a careful balance between protecting these probiotics from the harsh vaginal environment and ensuring their pH-responsive release. This study proposes a rational design of a CMCS-OHA-based hydrogel system tailored to the vaginal delivery of Lactobacillus species (Scheme 1). This platform is engineered to optimize the dual functionality of probiotic protection against vaginal environmental stressors and the pH-responsive release of the encapsulated probiotics, thereby modulating the vaginal microecological environment and combating *C. albicans* infection.

## 2. Materials and Methods

### 2.1. Materials

Carboxymethyl chitosan (CMCS, degree of carboxylation ≥ 85%), hyaluronic acid (HA, Mw = 10–20 kDa), sodium periodate (NaIO_4_), ethylene glycol (EG), and phosphate buffer solution (PBS) were purchased from McLean Biochemical Technology Co., Ltd. (Shanghai, China). Crystal purple and live/dead fluorescence kit were purchased from Biyuntian Biotechnology Co., Ltd. (Shanghai, China). Dimethyl sulfoxide (DMSO) was purchased from Taitan Technology Co., Ltd. (Shanghai, China). High-glucose DMEM culture medium, penicillin–streptomycin, fetal bovine serum, and 0.25% trypsin were purchased from Haotian Biotechnology Co., Ltd. (Hangzhou, China). Human vaginal epithelial cells (VK2 E6E7) were purchased from China Typical Culture Preservation Center (Shanghai, China). *L. plantarum* (Lp), *L. rhamnosus* (Lr), and *C. albicans* were all purchased from China Microbiological Culture Preservation Center (Beijing, China), and 4% paraformaldehyde was purchased from Bode Bioengineering Co., Ltd. (Wuhan, China). Female Sprague–Dawley (SD) rats aged 6–8 weeks were purchased from Hangzhou Hangsi Biotechnology Co., Ltd. (Hangzhou, China). Other reagents used in this experiment were of analytical grade or higher.

### 2.2. Preparation of CMCS, OHA, CMCS-OHA, and CMCS-OHA-Lp/Lr Hydrogels

The OHA was prepared through sodium periodate oxidation [48]. In a beaker with 50 mL of distilled water, 2.00 g of HA was dissolved, heated to 37 °C in a water bath, and swirled at 600 rpm until it was fully dissolved. After cooling to room temperature, 1.30 g of NaIO_4_ (1.30 g) was added. The mixture was continuously stirred in a 25 °C water bath for 12 h and then protected from light. The unreacted sodium periodate was quenched by adding EG (1 mL) and stirring for an additional hour. The product was obtained after dialysis and freeze-drying.

CMCS was dissolved in PBS 7.4 to create a uniform solution with a mass fraction (*w*/*v*) of 5%. Similarly, homogeneous solutions with mass fractions (*w*/*v*) of 3%, 4%, and 5% were created by dissolving the produced OHA in PBS 7.4. Equal volumes of the OHA and CMCS solutions were mixed until a gel was formed. The resulting blank hydrogels were named CMCS-OHA (5–3%), CMCS-OHA (5–4%), and CMCS-OHA (5–5%), respectively. Similarly, CMCS-OHA (1:1), CMCS-OHA (1:2), and CMCS-OHA (2:1) were obtained by mixing varying volumes of OHA and CMCS solutions.

MRS medium, Petri dishes and freeze-dried bacteria tubes were pre-sterilized and placed on the ultra-clean bench. The alcohol lamp was lit, and the sealed end of the bacterial ampoule was flamed until red-hot. An aseptic pipette was then employed to aspirate a small amount of liquid medium, which was deposited onto the heated ampoule seal. Upon cooling, the seal burst. A fresh aseptic pipette tip used to absorb an appropriate amount of MRS liquid medium, which was then added to the freeze-dried bacteria tube. The mixture was vortex-mixed for complete suspension and subsequently transferred to the liquid medium. In addition, the bacterial suspension was streaked and inoculated on MRS agar slant medium using an inoculation loop. Inoculated liquid medium and agar slant medium were cultured in an incubator for 24 h. Lyophilized *L. plantarum* (Lp) and *L. rhamnosus* (Lr) were sub cultured 2–3 times. After 5 min of centrifuging at 5000 rpm, the suspensions were rinsed with sterile PBS 7.4. The washed *L. plantarum* (Lp) and *L. rhamnosus* (Lr) were re-suspended and adjusted to the appropriate density (10^8^ CFU). CMCS-OHA-Lp and CMCS-OHA-Lr hydrogels were successfully prepared by dissolving OHA powder in the aforementioned PBS solution and mixing it evenly with CMCS solution in a water bath at 37 °C until it formed a gel [39].

### 2.3. Characterization of Hydrogels—Fourier Transform Infrared Spectrum, ^1^H NMR Analysis, and SEM

The infrared spectra of HA, OHA, CMCS, and CMCS-OHA hydrogels were acquired in the 4000–400 cm⁻^1^ range using an FT-IR (Nicolet 6700) spectrometer [49]. ^1^H NMR spectra of HA and OHA were recorded at 25 °C using D_2_O as a solvent on an AVANCE III 400 MHz Bruker instrument. Hydrogels were freeze-dried (LGJ-10) and coated with gold in order to examine their morphology using field emission scanning electron microscopy (FEI QUTAN FEG 250) [50]. The ImageJ 1.8.0 Image program was used to measure the hydrogel samples’ aperture. Images were taken from different areas of the hydrogel for each sample, and each picture contained more than five holes.

### 2.4. Characterization of Hydrogels—Rheological Test

A rheometer (DHR-1) was used to assess the rheological characteristics of hydrogels. A homogenous combination of 400 µL of OHA and CMCS was placed between parallel plates that had a 20 mm diameter and a 1000 µm gap. The energy storage modulus (G′) and loss modulus (G″) of the hydrogel system were measured at 37 °C with a 1 Hz frequency and 1% strain in order to assess the mechanical characteristics of the system.

### 2.5. Characterization of Hydrogels—Shear-Thinning and Self-Healing

The OHA was dissolved in a PBS solution (7.4) containing crystal violet at a concentration of 0.002% (*w*/*v*) and prepared as a uniform solution with mass fractions of 3%, 4%, and 5%, respectively. A 300 µL solution of CMCS (5%) was selected and mixed with the OHA solution to create a homogeneous mixture. Then, 300 μL of CMCS (5%) was uniformly mixed with the same volume of OHA solution in a water bath at 37 °C. Once the mixture reached a gel state, it was placed in a syringe (10 mL, 0.7 × 32 TWLB) to assess whether the hydrogel could be extruded through the needle without clogging [51]. Additionally, a rheometer was employed to evaluate the shear-thinning properties of the hydrogels. The viscosity of the different hydrogels was measured. The self-healing behavior of the CMCS-OHA hydrogel was assessed by reintegration testing of split pieces.

### 2.6. Characterization of Hydrogels—In Vitro Degradation Test

To determine gel degradation in vitro, CMCS-OHA, CMCS-OHA-Lp (10^8^ CFU), and CMCS-OHA-Lr (10^8^ CFU) were immersed in 8 mL PBS buffer solutions with pH values of 7.4 and 5.5, respectively, and the hydrogel blocks oscillated at 37 °C at 120 rpm. The volume of hydrogels used in this part was 600 μL. The hydrogels were removed at predetermined intervals, and any remaining water on the surface was weighed after being absorbed with absorbent paper. The weight remaining rate (%) of a hydrogel is defined by the following formula:

(1) $$\mathrm{Weight}\;\mathrm{remaining}\;\mathrm{ratio}\;(\%)\;\mathrm{of}\;\mathrm{hydrogels}\;(\%)\;=\;\frac{W_{t}}{W_{0}}\times 100\%$$

where *W_t_* and *W*_0_ represent the weight of residual hydrogel and the weight of original hydrogel, respectively.

### 2.7. Release of Lactobacillus In Vitro

Initially, 1 mL of hydrogel (CMCS-OHA-Lp, CMCS-OHA-Lr) loaded with *L. plantarum* (10^8^ CFU) and *L. rhamnosus* (10^8^ CFU) was immersed in 4 mL of sterile PBS buffer with pH 5.5 (vaginal microenvironment) and 7.4 (physiological environment), respectively. The samples were incubated with an oscillating speed of 120 rpm at 37 °C. At specific time intervals (0, 4, 8, 12, 24, 36, and 48 h), the incubated PBS solution (7.4) was diluted in a 10-fold series to 10^−2^ or 10^−3^. MRS agar plates were cultivated for 48 h at 37 °C [52]. Each group conducted three parallel experiments. The Lactobacillus release rate is defined as follows:

(2) $$\mathrm{Release}\;\mathrm{rate}\;(\%)=\;\frac{N_{t}\times 20\times dilutability\times V}{N}\times 100\%$$

where *N* and *N_t_* represent the total number of colonies and the number of colonies at different times, respectively, and V represents the volume of the releasing medium.

### 2.8. Antifungal Experiment In Vitro

#### 2.8.1. Antifungal Zone Experiment

Initially, 1 mL of diluted *C. albicans* suspension was added to 200 mL of Sabouraud Dextrose Agar (SDA), which had been cooled to approximately 50 °C. The mixture was thoroughly mixed and poured into plates, and then set horizontally for future use. Oxford cups were placed horizontally on the agar surface. Subsequently, 100 µL CMCS-OHA, CMCS-OHA-Lp (10^8^ CFU), CMCS-OHA-Lr (10^8^ CFU), and Lp and Lr bacterial suspension were injected into the Oxford cup in succession. The same volume of sterile PBS solution (7.4) was used as the control group. The experimental plates were incubated for 24 h at 37 °C. The antifungal zone’s diameter was measured to determine how effective the treatment was [53,54].

#### 2.8.2. Plate Counting Method

Initially, 500 µL of CMCS-OHA-Lp, CMCS-OHA-Lr, CMCS-OHA hydrogel, and Lp and Lr bacterial suspensions (10^8^ CFU) were added to sterile 24-well plates. The same volume of sterile PBS solution (7.4) was used as the control group. Each well was then filled with 10 µL of diluted *C. albicans* suspension (10^8^ CFU/mL) and 3 mL of liquid medium. The plates were kept in a shock culture incubator set at 37 °C for a whole day. After incubation, 50 µL of the co-culture solution was aspirated. The co-culture solution was diluted continuously by the gradient dilution method. The appropriate dilution concentrations were spread evenly onto the Sabouraud Dextrose Agar (SDA), and viable fungal colonies were counted after incubating at 37 °C for 24 h. The following formula was used to obtain the inhibition rate:

(3) $$\mathrm{Inhibition}\;\mathrm{Rate}\;(\%)=\frac{N_{0}-N_{1}}{N_{0}}\times 100\%$$

The number of colonies on the experimental group plate is *N*_1_, while the number on the blank group plate is *N*_0_. The experiment consisted of three parallel experiments (*n* = 3).

### 2.9. Biofilm Inhibition Experiment

The crystal violet staining method was employed to quantify the biofilm [54,55]. Specifically, 200 µL of *C. albicans* suspension (10^8^ CFU/mL) was added to the 96-well plate and placed in a 37 °C constant-temperature incubator for 48 h. After biofilm formation, 200 µL of Lp and Lr suspensions (10^8^ CFU/mL, 10^7^ CFU/mL) and CMCS-OHA, CMCS-OHA-Lp, and CMCS-OHA-Lr (10^8^ CFU/mL and 10^7^ CFU/mL) were added at varying concentrations. The same volume of sterile PBS solution was used as the control group. Salle’s medium was given to the control group in the same volume, and they were incubated for 24 h at 37 °C. After removing the medium, sterile PBS 7.4 was used to rinse the biofilm three times. Following a 30 min fixation period, 100 µL of methanol was applied; the methanol was then disposed of. Following the addition of 100 µL of 1% crystal violet solution for staining, the biofilm was rinsed three times with sterile PBS 7.4 and the crystal violet solution was disposed of after 15 min. After that, the sample was allowed to air dry at the normal temperature. Then, 200 µL of 95% ethanol was added to each well and an ELISA plate reader was used. Biofilm elimination was calculated as follows:

(4) $$\mathrm{Biofilm}\;\mathrm{elimination}\;\mathrm{Rate}\;(\%)=\;\frac{OD_{0}-OD_{1}}{OD_{0}}\times 100\%$$

*OD*_0_ and *OD*_1_ represent the absorbance of the control group and the experimental group, respectively.

### 2.10. Storage Activity Test

Lactobacillus activity was determined using the plate counting method. First, 500 µL of CMCS solution was added to a centrifuge tube. Bacterial suspensions of Lp and Lr with a concentration of 10^10^ CFU/mL were prepared and then suspended centrifugally. Then, 500 µL of the previously stated bacterial solutions was used to dissolve the OHA polymer. The centrifuge tube was then filled with this solution to create the CMCS-OHA-Lp and CMCS-OHA-Lr hydrogels. Suspensions of Lp and Lr with the same colony concentration were used as the control group and stored at 4 °C and 25 °C for 0, 5, 10, 20, and 30 days, respectively. At specific time points, sterile PBS with pH 5.5 was added to fully degrade the hydrogel and release the Lactobacillus. Sterilized PBS was used to dilute 50 µL of the hydrogel solution to the proper concentration. For colony counting, 50 µL of the diluted solution was then applied to MRS solid agar medium and cultured for 48 h at 37 °C. The formula for calculating the activity retention rate is as follows:

(5) $$\mathrm{Activity}\;\mathrm{retention}\;\mathrm{Rate}\;(\%)=\;\frac{M}{M_{0}}\times 100\%$$

where *M* represents the pair value of active Lactobacillus (CFU) in the hydrogel live bacterial suspension at a specific time, and *M*_0_ is the pair value of the total active Lactobacillus (CFU).

### 2.11. Cytocompatibility

#### 2.11.1. Cytotoxicity Evaluation of the Hydrogel (CMCS-OHA)

LIVE/DEAD^®^ kits and MTT assays were used to assess cell viability in the presence of hydrogels. Human vaginal epithelial cells (VK2 E6E7) were seeded onto 96-well plates at a density of 5 × 10^3^ cells per well. The plates were then incubated at 37 °C with 5% CO_2_ for the whole night. Hydrogel extracts at different quantities (2.5 mg/mL, 1.25 mg/mL, and 0.625 mg/mL) were then added to the medium. The negative control was an extract that was not substituted, and the blank control was pure culture medium. Each well received 10 µL of MTT (5 mg/mL, dispersed in sterile PBS) after being incubated for 24, 48, and 72 h. The wells were then incubated for 4 h. Following the removal of the MTT solution, 150 µL of DMSO was applied to each well. Fluorescence was measured using an enzyme-labeled meter at 490 nm excitation and 570 nm emission. A microplate reader was used to record the fluorescence intensity of each well in order to evaluate cell viability. The following formula was used to determine cell viability:

(6) $$\mathrm{Cell}\;\mathrm{viability}\;(\%)=\;\frac{ODexperiment-ODblank}{ODnegative-ODblank}\times 100\%$$

VK2 E6E7 were injected into plates with 24 wells at a count of 10^5^ cells per well. After adding one milliliter of high-glucose DMEM media, the plates were incubated for the entire night at 37 °C with 5% CO_2_. Hydrogels with volumes of 500 μL, 250 μL, and 125 μL were immersed in 10 mL high-glucose DMEM medium for 24 h (37 °C, 5% CO_2_), and the hydrogel extracts with different concentrations were obtained. Following incubation, 1 mL of hydrogel extract (2.5 mg/mL, 1.25 mg/mL, and 0.625 mg/mL) was added after the medium was withdrawn. The extracts were not replaced for the negative control group. Following incubation for 24, 48, and 72 h, the extracts were removed, and the wells were washed 1–2 times with sterile PBS. The live/dead cell staining kit’s instructions were followed for performing the cell staining. Next, using a fluorescent microscope (BX 43, Olympus, Tokyo, Japan), the vitality and adherence of the cells were examined.

#### 2.11.2. Cytotoxicity Evaluation of CMCS-OHA-Lr and CMCS-OHA-Lp

The experimental procedure is detailed in Section 2.11.1. The concentrations were set to CMCS-OHA-Lp (10^7^, 10^8^, and 10^9^ CFU) and CMCS-OHA-Lr (10^7^, 10^8^, and 10^9^ CFU).

### 2.12. Establishment of VVC Model and Antifungal Effect In Vivo

The National Research Council Guidelines for the Care and Use of Laboratory Animals and the Zhejiang University of Technology Guidelines for the Care and Use of Laboratory Animals in Hangzhou, China were followed in all animal testing protocols. For the study, female SD rats weighing 180–220 g and 6–8 weeks of age were used. Prior to infection, the animals received a subcutaneous injection of 0.2 mL (2 mg/mL) of estradiol benzoate solution [56]. Starting on the third day, the vaginas of the rats were irrigated with sterile PBS, and the presence of enucleated epithelial cells in the irrigation solution was observed under a microscope. A 100 µL suspension of *C. albicans* (10^9^ CFU/mL) was injected into the vaginas of the rats daily from the onset of spontaneous estrus [57]. Healthy rats were injected intravaginally with 100 µL of sterile PBS 7.4 for 2 consecutive days. To verify a successful *C. albicans* infection, 50 µL of secretions were also seeded onto agar medium at a particular time point, and the optical density (OD) at 600 nm was measured using a multi-well microplate reader.

The rats were randomly divided into seven groups: (1) healthy group, (2) control group (model group), (3) CMCS-OHA hydrogel group, (4) Lp bacterial suspension (10^8^ CFU) group, (5) Lr bacterial suspension (10^8^ CFU) group, (6) CMCS-OHA-Lp (10^8^ CFU) hydrogel group, and (7) CMCS-OHA-Lr (10^8^ CFU) hydrogel group. Each group consisted of six rats. Beginning on the fourth day, the rats in the experimental groups received intravaginal treatments (200 µL) once daily for five days in a row. The healthy group and the negative control group were not treated and were fed normally. After 0, 3, and 6 days of treatment, the vagina was irrigated with sterile PBS 7.4, it was diluted through a series of ten-fold dilutions. CFUs were then measured after 50 µL of the diluted solution was placed onto solid agar medium and cultured for 48 h at 37 °C.

### 2.13. In Vivo Safety Analysis

The in vivo safety of the hydrogel system was analyzed by weight change curves and H&E staining sections. SD rats were randomly assigned to six groups: an untreated group, a CMCS-OHA hydrogel group, a CMCS-OHA-Lp (10^8^ CFU) hydrogel group, a CMCS-OHA-Lr (10^8^ CFU) hydrogel group, an Lp bacterial suspension group, and an Lr bacterial suspension group (10^8^ CFU). It is noteworthy that none of the SD rats underwent molding, and all were euthanized after five days of continuous administration. After the experiment, the heart, liver, spleen, lungs, kidneys, and vaginal tissues were collected, soaked in a 4% paraformaldehyde solution for one hour, and sectioned into 4 µm slices using a microtome. The sections were stained with H&E stain.

### 2.14. Vaginal Tissue Inflammation and Immunohistochemical Analysis

The changes in vaginal inflammation among SD rats in each group were analyzed using H&E staining. SD rats from each group were euthanized at the end of the trial, and the vaginal tissues were taken, immersed for an hour in a 4% paraformaldehyde solution, and cut into 4 µm slices with a microtome. Histopathological changes were observed under a microscope. Additionally, the sections were evaluated through immunohistochemical analysis to assess the degree of inflammation.

### 2.15. Statistical Analysis

GraphPad Prism version 8.0 was used to preprocess the data. Statistical analyses were conducted using SPSS statistical software version 19.0 (SPSS Inc., Chicago, IL, USA), employing T-tests and one-way analysis of variance. The mean ± standard deviation is used to express any quantitative data. The threshold for statistical significance was set at *p* < 0.05. The legend displays the following degrees of statistical significance: NS: no significant change; * *p* < 0.05, ** *p* < 0.01, *** *p* < 0.001, and **** *p* < 0.0001.

## 3. Results

### 3.1. Preparation and Characterization of CMCS-OHA Hydrogel

**Figure 1:**
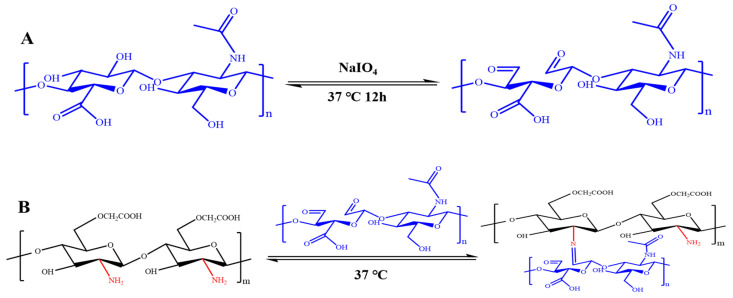
CMCS-OHA hydrogel preparation. (**A**,**B**) Schematic representation of the synthesis of OHA and CMCS-OHA hydrogels.

The synthesis of the CMCS-OHA hydrogel was successfully achieved, as demonstrated by the cross-linking mechanism depicted in Figure 1A,B and confirmed through FT-IR analysis (Figure S1A) and ^1^H NMR (Figure S1B). Initially, OHA was synthesized, a process verified by the ^1^H NMR analysis, which detected the N-acetyl methyl protons of HA and OHA at 2.0 ppm. A broad signal between 3.2 and 3.7 ppm was observed, corresponding to various protons in the sugar ring. Notably, new signal peaks at 4.9, 5.0, and 5.1 ppm in OHA (chemical potential shift towards higher field due to the formation of hydration) indicated the successful oxidation of HA.

Attenuated total reflection (ATR) mode FT-IR analysis was used to investigate the surface chemistry of CMCS, OHA, and CMCS-OHA hydrogels (Figure S1A). The antisymmetric stretching vibration of -COOH, the stretching absorption of the C-O bond, and the absorption of -OH were identified as the causes of the distinctive peaks of HA at 3400, 1616, and 1078 cm⁻^1^, respectively. With the exception of a tiny band at 1730 cm⁻^1^ that corresponded to the aldehyde group and was in agreement with the ^1^H NMR results, the OHA spectra was quite similar to that of HA. In the CMCS spectrum, the peak at 1411 cm⁻^1^ was associated with the symmetric contraction of the carboxyl group (-COO^−^). The spectra of CMCS-OHA 1:1 and CMCS-OHA 2:1 hydrogels exhibited all the characteristic bands of CMCS and OHA. Crucially, the aldehyde group peak at 1730 cm⁻^1^ diminished or vanished, indicating that the amino group in CMCS formed an imine bond with the aldehyde group in OHA, causing gel formation. As the proportion of CMCS increased, the aldehyde group in OHA completely reacted to form the imine bond, leading to the disappearance of the signal peak. This observation is consistent with the proposed cross-linking mechanism of CMCS-OHA hydrogels.

### 3.2. Rheological, Shear-Thinning, and Self-Healing Properties of CMCS-OHA Hydrogels

**Figure 2:**
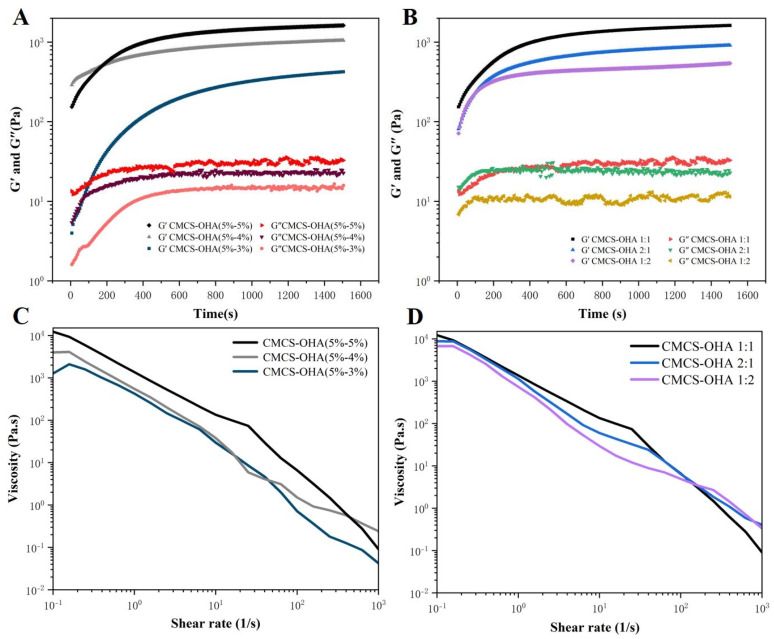
The mechanical and shear-thinning of the CMCS-OHA hydrogel were characterized. (**A**) G′ and G″ curves for CMCS-OHA (5–3%), CMCS-OHA (5–4%), and CMCS-OHA (5–5%) over time. (**B**) G′ and G″ curves for CMCS-OHA (1:1), CMCS-OHA (2:1), and CMCS-OHA (1:2) over time. (**C**) Shear–viscosity curves for CMCS-OHA (5–3%), CMCS-OHA (5–4%), and CMCS-OHA (5–5%). (**D**) Viscosity–shear curves for CMCS-OHA (1:1), CMCS-OHA (2:1), and CMCS-OHA (1:2).

The gelation process and mechanical characteristics of mixed aqueous solutions of CMCS and OHA were observed in situ using a rheometer. The rapid cross-linking between the -CHO and -NH_2_ groups forms a dynamic Schiff base to form gelation (Figure S1), resulting in the energy storage modulus (G′) rapidly exceeding the loss modulus (G″) and reaching a maximum value (Figure 2A). To evaluate the effects of varying OHA concentrations on the mechanical properties of the hydrogels, measurements of G′ and G″ were conducted for CMCS-OHA (5–3%), CMCS-OHA (5–4%), and CMCS-OHA (5–5%) (Figure 2A). All CMCS-OHA hydrogels demonstrated a high-energy storage modulus (G′), indicative of their ability to form stable cross-linked networks. Notably, CMCS-OHA (5–5%) hydrogels demonstrated the highest G′, approximately 2 × 10^3^ Pa, significantly exceeding that of the other CMCS-OHA hydrogels. This increase is attributed to the higher concentration of OHA, which enhances the proportion of -CHO groups, thereby increasing the number of Schiff base bonds and resulting in a denser polymer network. With the OHA concentration fixed at 5%, further analysis of the G′ and G″ was performed for CMCS-OHA (1:1), CMCS-OHA (2:1), and CMCS-OHA (1:2) (Figure 2B). All hydrogels displayed a high-energy storage modulus (G′), with CMCS-OHA (1:1) hydrogels exhibiting the highest G′, around 2 × 10^3^ Pa. Considering both gelation time and mechanical strength, the CMCS-OHA (1:1) hydrogel was selected as the focus of our research, maintaining the OHA concentration at 5%.

The shear-thinning properties of the CMCS-OHA hydrogel were confirmed through rheological and needle extrusion tests. All CMCS-OHA hydrogels showed shear-thinning characteristics. It is characterized by a decrease in viscosity with the increase in shear force. Specifically, as the shear rate increased from 10^−1^ 1/s to 10^3^ 1/s, the viscosity of the CMCS-OHA hydrogel decreased from 10^4^ Pa·s to 10^−1^ Pa·s (Figure 2C,D). In addition, when the hydrogel was loaded into a syringe system (Figure S1), it could be extruded through the needle and subsequently regained its shape in various forms after injection (Figure S1), demonstrating excellent shear-thinning properties. These characteristics enhance the delivery of Lactobacillus within the body, alleviating discomfort and promoting faster recovery for patients. Using the CMCS-OHA (1:1) hydrogel as an example, when four pieces of hydrogel with alternating colors were cut and recombined, the boundary between the two different colors became blurred and merged into a single entity, indicating molecular movement between adjacent hydrogel pieces (Figure S1). Even when tension was applied to stretch the healed hydrogel, the previously separated sections remained intact. These findings collectively suggest that CMCS-OHA hydrogels possess remarkable self-healing capabilities, with the primary mechanism of self-healing being the dynamic interactions between functional groups rather than mere physical adhesion at the fractured hydrogel interface.

### 3.3. Morphology and pH-Dependent Degradation of CMCS-OHA and CMCS-OHA-Lp/Lr In Vitro

**Figure 3:**
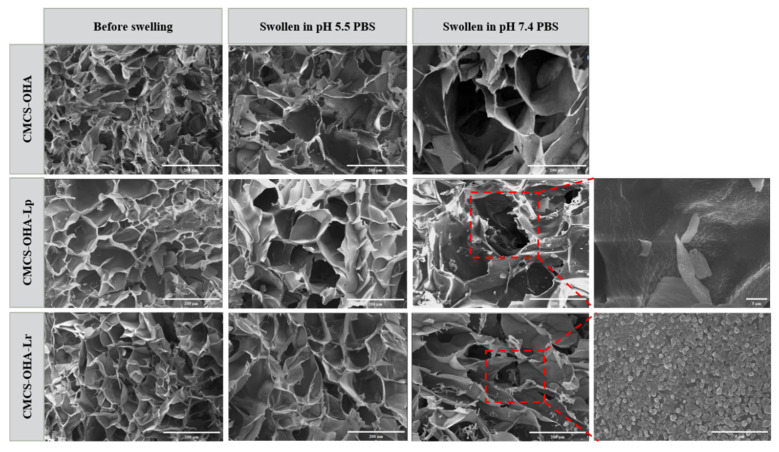
SEM images of CMCS-OHA (1:1), CMCS-OHA-Lp (10^8^), and CMCS-OHA-Lr (10^8^) hydrogels before and after swelling; scale bar: 200 µm. The locally enlarged image illustrates the morphology of Lactobacillus within the gel; scale bar: 5 µm, 10 um.

**Figure 4:**
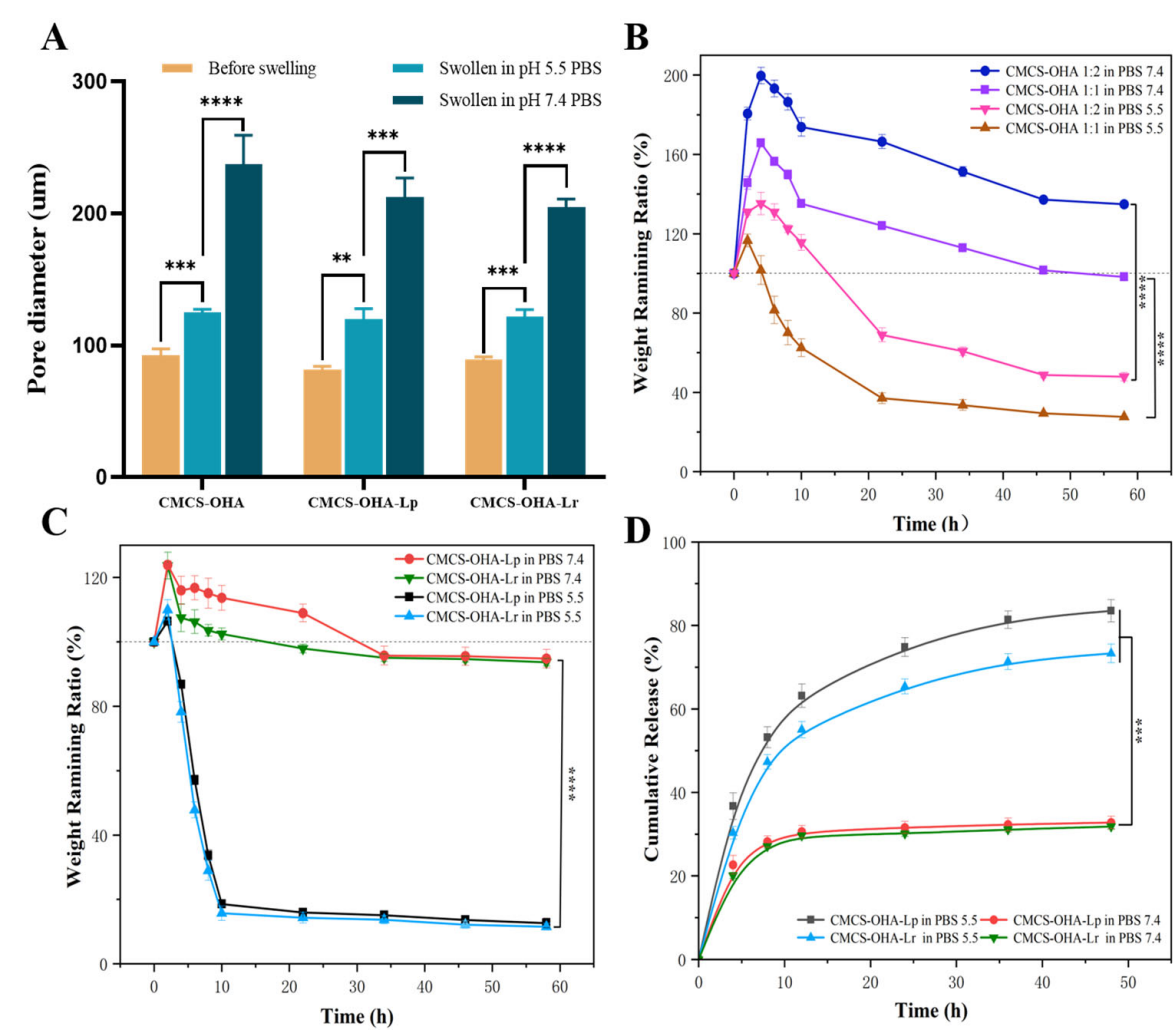
Pore size, swelling, and degradation characteristics of CMCS-OHA, CMCS-OHA-Lp, and CMCS-OHA-Lr hydrogels, along with the release of Lactobacillus. (**A**) Average pore size of the hydrogels before and after swelling (*n* = 5). (**B**) Degradation curves of CMCS-OHA (1:1) and CMCS-OHA (1:2) in pH 7.4 and 5.5 PBS (*n* = 3). (**C**) Degradation curves of CMCS-OHA-Lp (10^8^) and CMCS-OHA-Lr (10^8^) in pH 7.4 and 5.5 PBS (*n* = 3). (**D**) Cumulative release curves of Lactobacillus from CMCS-OHA-Lp (10^8^) and CMCS-OHA-Lr (10^8^) hydrogels in vitro (*n* = 3). The results represent the mean ± SD. (** *p* < 0.01, *** *p* < 0.001, and **** *p* < 0.0001).

The microstructures of the hydrogels were analyzed using scanning electron microscopy (SEM), focusing on their pore sizes. As shown in Figure 3 and Figure 4A, all hydrogels exhibited uniform and interconnected porous structures. Specifically, the CMCS-OHA hydrogel system demonstrated variations in pore sizes before and after swelling in PBS at different pH levels. The pore size distribution of the CMCS-OHA hydrogel system both pre- and post- swelling in PBS at varying pH levels is depicted in Figure 4A. Before swelling, the pore sizes of CMCS-OHA, CMCS-OHA-Lp, and CMCS-OHA-Lr hydrogels were approximately 93 ± 5 µm, 82 ± 2 µm, and 89 ± 2 µm, respectively.

Upon swelling in PBS at pH 5.5, these pore sizes increased to about 125 ± 3 µm, 120 ± 8 µm, and 122 ± 5 µm, respectively. In contrast, swelling in PBS at pH 7.4 resulted in significantly larger pore sizes of approximately 237 ± 22 µm, 213 ± 14 µm, and 205 ± 6 µm. Notably, the pore sizes of CMCS-OHA-Lp and CMCS-OHA-Lr hydrogels were slightly smaller than those of the standard CMCS-OHA hydrogels, suggesting the successful encapsulation of Lactobacillus. The smaller pore size of CMCS-OHA hydrogels in acidic environments compared to neutral ones is likely due to partial degradation caused by the fracture of Schiff base bonds in acidic conditions.

In order to better investigate the pH-dependent degrading behavior of CMCS-OHA, CMCS-OHA-Lp, and CMCS-OHA-Lr hydrogels, we carried out tests in physiological (PBS, pH 7.4) and acidic (PBS, pH 5.5) microenvironments. As illustrated in Figure 4B, hydrogels in PBS at pH 5.5 degraded significantly faster than those in PBS at pH 7.4, with CMCS-OHA 1:1 showing a notable difference. After 24 h, the mass of CMCS-OHA 1:1 in PBS at pH 5.5 decreased to approximately 37% and further reduced to 28% after 58 h, whereas in PBS at pH 7.4, the mass remained at 98%, indicating minimal degradation. CMCS-OHA-Lp and CMCS-OHA-Lr hydrogels exhibited even more rapid and complete degradation in acidic environments, as shown in Figure 4C. After just 10 h in PBS at pH 5.5, the masses of CMCS-OHA-Lp and CMCS-OHA-Lr were reduced to 19% and 16%, respectively, while they showed negligible degradation in PBS at pH 7.4. The accelerated degradation of CMCS-OHA-Lp and CMCS-OHA-Lr hydrogels compared to CMCS-OHA hydrogels can be attributed to lactic acid production by Lactobacillus, which lowers the pH and facilitates the breakdown of Schiff base bonds. These findings underscore the high sensitivity of the CMCS-OHA hydrogel system to acidic conditions, highlighting its pH-dependent degradation characteristics and potential for pH-responsive release of Lactobacillus.

### 3.4. In Vitro Release of Lactobacillus from CMCS-OHA-Lp/Lr

The pH-responsive drug release characteristics of CMCS-OHA-Lp and CMCS-OHA-Lr hydrogels were examined in physiological environments (PBS, pH 7.4) and vaginal microenvironments (PBS, pH 5.5) (Figure 4D). The release profiles for both Lactobacillus species demonstrate an initial rapid release phase, irrespective of whether the pH is 7.4 or 5.5. This swift release can be attributed to the immediate penetration of PBS into the hydrogel, which facilitates the relaxation of the hydrogel’s molecular chains and consequently the rapid release of Lactobacillus. Additionally, the rapid release may also result from Lactobacillus being distributed on the hydrogel surface. At pH 5.5, the cumulative release rates for *L. plantarum* (Lp) and *L. rhamnosus* (Lr) reached 83.5 ± 2.70% and 73.3 ± 2.2%, respectively, within 48 h, suggesting that the release process had nearly reached equilibrium. Conversely, at pH 7.4, the cumulative release rates for *L. plantarum* (Lp) and *L. rhamnosus* (Lr) were significantly lower, at 32.8 ± 1.5% and 31.8 ± 0.7% after 48 h. These findings indicate that under the acidic conditions of the vaginal microenvironment (pH 5.5), the hydrogels exhibit pH-responsive release behavior, and the hydrogel matrix is capable of degrading in response to the acidic environment.

### 3.5. Antifungal Experiment In Vitro

**Figure 5:**
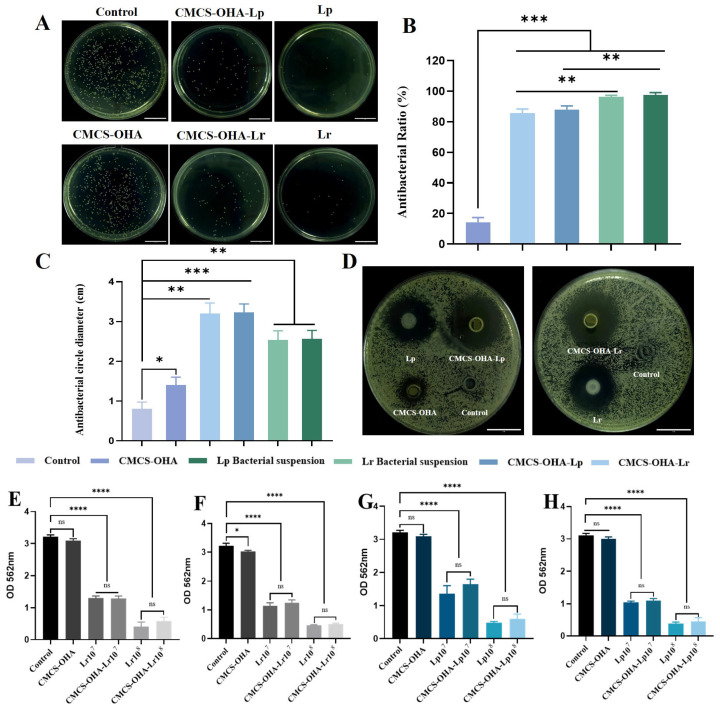
In vitro antifungal activities and anti-biofilm effects of CMCS-OHA (1:1), CMCS-OHA-Lp, and CMCS-OHA-Lr hydrogels, Lp and Lr suspensions, and control (PBS). (**A**) Colony of *C. albicans*, scale bar: 1 cm. (**B**) Antifungal rate (*n* = 3). (**C**) Quantitative analysis of antifungal zone diameter (*n* = 3). (**D**) Antifungal zone, scale bar: 1 cm. (**E**,**F**) Lr and CMCS-OHA-Lr inhibited the biofilm formation activity of *C. albicans* at 24 h and 48 h (*n* = 3). (**G**,**H**) Lp and CMCS-OHA-Lp on the biofilm formation activity of *C. albicans* at 24 h and 48 h (*n* = 3). The results represent the mean ± SD. (ns *p* > 0.05 * *p* < 0.05, ** *p* < 0.01, *** *p* < 0.001, and **** *p* < 0.0001).

The antifungal effect of the hydrogel was assessed using colony counting and the antifungal zone method. As depicted in Figure 5A, both CMCS-OHA-Lp and CMCS-OHA-Lr hydrogels, along with their respective bacterial suspensions, existed significant antifungal activity. Notably, the number of colonies in the CMCS-OHA hydrogel group did not show a significant reduction compared to the control group. However, the colony counts in the CMCS-OHA-Lp and CMCS-OHA-Lr hydrogel groups were significantly lower, indicating that the antifungal effect of the hydrogel was enhanced by the addition of Lactobacillus. In Figure 5B, the antifungal rates of the CMCS-OHA-Lp and CMCS-OHA-Lr hydrogel groups, as well as the Lp and Lr bacterial suspension groups, all exceeded 80%, demonstrating the hydrogel’s strong antifungal activity and its potential to effectively prevent fungal infections. Notably, the bacterial suspension group’s antifungal rate was marginally greater than the hydrogel group’s, which might be because Lactobacillus was partially inactivated following hydrogel coating or partially released. As shown in Figure 5C, compared to the CMCS-OHA hydrogel group, the antifungal zones of the CMCS-OHA-Lp and CMCS-OHA-Lr hydrogel groups were more distinct. Additionally, the CMCS-OHA-Lp and CMCS-OHA-Lr hydrogel groups’ antifungal zone diameters were noticeably larger than the CMCS-OHA hydrogel group (Figure 5D), suggesting that the addition of Lactobacillus significantly increased the CMCS-OHA hydrogel’s antifungal activity in accordance with the earlier colony counting results. Additionally, there was no significant difference in the antifungal effect between the CMCS-OHA-Lp and CMCS-OHA-Lr hydrogels.

Research indicated that biofilm colonization is a significant source of *C. albicans* virulence, making the inhibition of biofilm formation crucial. The inhibition of mature biofilm (Figure 5E,H) was employed to evaluate the antifungal effects of Lactobacillus. The CMCS-OHA-Lp and CMCS-OHA-Lr hydrogel groups showed concentration-dependent inhibition and an inhibitory effect comparable to that of the bacterial suspension group. As shown in Figure 5E–H, the control group formed a dense and complete biofilm with an optical density (OD) value greater than 3. After co-incubation with CMCS-OHA-Lp, CMCS-OHA-Lr hydrogel, and bacterial suspension with varying bacterial loads for 24 h, the biofilms of the CMCS-OHA-Lp10^8^ and CMCS-OHA-Lr10^8^ hydrogel groups were barely stained, exhibiting OD values of less than 1. After 48 h of co-incubation, the optical density (OD) values of the CMCS-OHA-Lp10^8^ and CMCS-OHA-Lr10^8^ hydrogel groups decreased, although this reduction was not statistically significant. This finding suggested that both hydrogel groups can significantly inhibit the formation of mature biofilms within 24 h, of which CMCS-OHA-Lp10^8^ and CMCS-OHA-Lr10^8^ align with previous results concerning adhesion inhibition. In conclusion, both CMCS-OHA-Lp10^8^ and CMCS-OHA-Lr10^8^ hydrogels demonstrated strong antifungal activity and effectively prevent fungal infections.

### 3.6. Storage Activity of Lactobacillus

**Figure 6:**
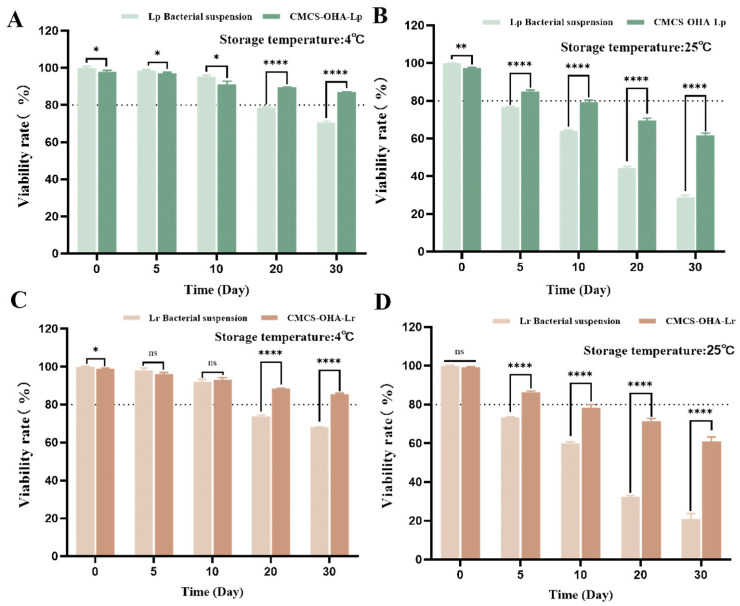
Lactobacillus activity of CMCS-OHA-Lp and CMCS-OHA-Lr hydrogels, as well as Lp and Lr bacterial suspensions, stored at 4 °C and 25 °C for 30 days (*n* = 3). (**A**) Storage activity of CMCS-OHA-Lp and Lp bacterial suspensions at 4 °C. (**B**) Storage activity of CMCS-OHA-Lp and Lp bacterial suspensions at 25 °C. (**C**) Storage activity of CMCS-OHA-Lr and Lr bacterial suspensions at 4 °C. (**D**) Storage activity of CMCS-OHA-Lr and Lr bacterial suspensions at 25 °C. The results represent the mean ± SD. (ns *p* > 0.05 * *p* < 0.05, ** *p* < 0.01, and **** *p* < 0.0001).

The maintenance of Lactobacillus activity is crucial for its therapeutic effects (Figure 6). At both storage temperatures, the effects of varying storage times and different formulations on the activity of the probiotics were significantly different. At a storage temperature of 4 °C, both free and gelled Lactobacillus exhibited a higher survival rate over 30 days (Figure 6A,C). This phenomenon may be attributed to the reduced metabolic activity and lower nutrient requirements of probiotics under cold conditions. After 30 days of storage at 4 °C, the survival rates of free Lp and Lr were 70.5 ± 0.9% and 68.2 ± 0.3%, while the survival rates of CMCS-OHA-Lp and CMCS-OHA-Lr were 86.9 ± 0.2% and 85.5 ± 0.6%, respectively.

These results indicate that hydrogel coating is beneficial for maintaining Lactobacillus activity to some extent. At a storage temperature of 25 °C, the number of viable Lactobacillus significantly decreased with prolonged storage time, particularly for free Lp and Lr, with survival rates after 30 days dropping to only 28.8 ± 1.1% and 21.1 ± 2.8%, respectively (Figure 6B,D). Although the survival rates of Lactobacillus in CMCS-OHA-Lp and CMCS-OHA-Lr also declined, they remained above 60%. These findings clearly demonstrate that hydrogel coating can effectively mitigate the impact of ambient temperature on the activity of probiotics, thereby extending their storage stability.

### 3.7. In Vitro Biocompatibility of the CMCS-OHA-Lp and CMCS-OHA-Lr Hydrogels

**Figure 7:**
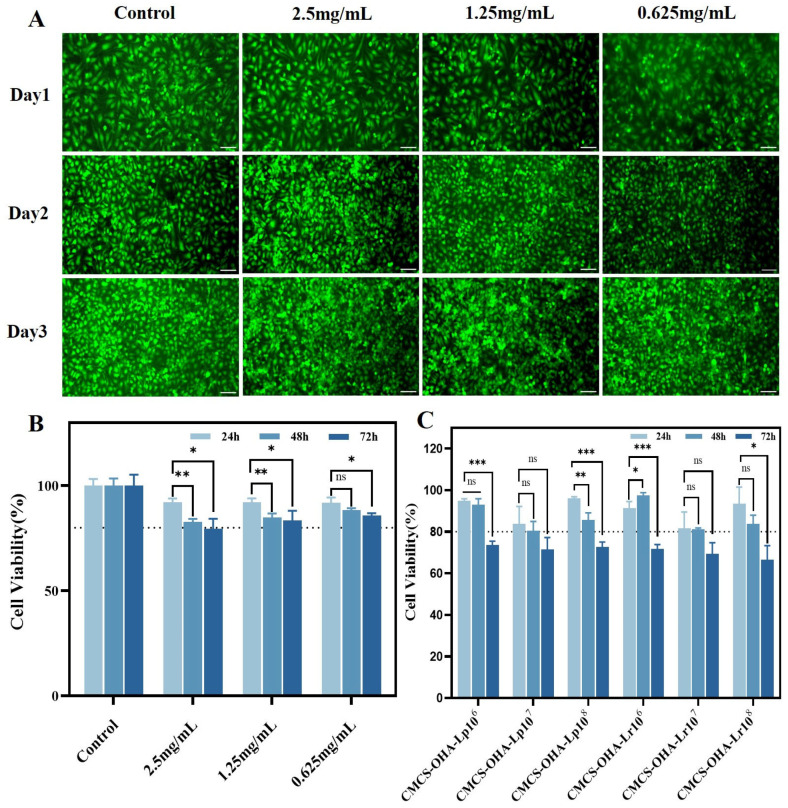
Cytocompatibility of CMCS-OHA, CMCS-OHA-Lp, and CMCS-OHA-Lr hydrogels. (VK2 E6E7). (**A**) VK2 E6E7 cell live/dead staining images in CMCS-OHA hydrogel extract at various stages of incubation. Scale bar: 50 µm. (**B**) MTT analysis of VK2 E6E7 cells at different concentrations in CMCS-OHA hydrogel extracts (*n* = 6). (**C**) MTT analysis of VK2 E6E7 cells in CMCS-OHA-Lp and CMCS-OHA-Lr with different bacterial loads (*n* = 6). The results represent the mean ± SD. (ns *p* > 0.05 * *p* < 0.05, ** *p* < 0.01, and *** *p* < 0.001).

To evaluate the biocompatibility of the developed CMCS-OHA hydrogel, we conducted a series of experiments involving VK2 E6E7 cells with results depicted in Figure 7A. Observations indicated an increase in cell density over time, indicating that the CMCS-OHA hydrogel did not adversely affect cell proliferation or attachment. To quantitatively evaluate the hydrogel’s impact, an MTT cell viability assay was conducted. As illustrated in Figure 7B, the hydrogel extracts had a minimal effect on the viability of VK2 E6E7 cells compared to the control group. Although cell viability slightly decreased over time, it remained above 80% in both cell types, indicating that the CMCS-OHA hydrogel was not cytotoxic. We examined the effects of CMCS-OHA hydrogels loaded with *L. plantarum* (Lp) and *L. rhamnosus* (Lr) on VK2 E6E7 cells using MTT assay. For VK2 E6E7 cells, the CMCS-OHA-Lp and CMCS-OHA-Lr hydrogels, with varying bacterial loading rates, maintained cell viability at approximately 80% over two days. However, VK2 E6E7 cells showed a slight decrease in viability to approximately 75% at 72 h (Figure 7C). This decline may be attributed to the production of lactic acid by Lactobacillus, leading to a low environmental pH that is less conducive to cell growth. In conclusion, CMCS-OHA-Lp and CMCS-OHA-Lr hydrogels, particularly CMCS-OHA-Lp10^8^ and CMCS-OHA-Lr10^8^, exhibit good biocompatibility. Consequently, these hydrogels were selected for further research due to their effective bacteria-carrying capacity.

### 3.8. In Vivo Pharmacodynamics

**Figure 8:**
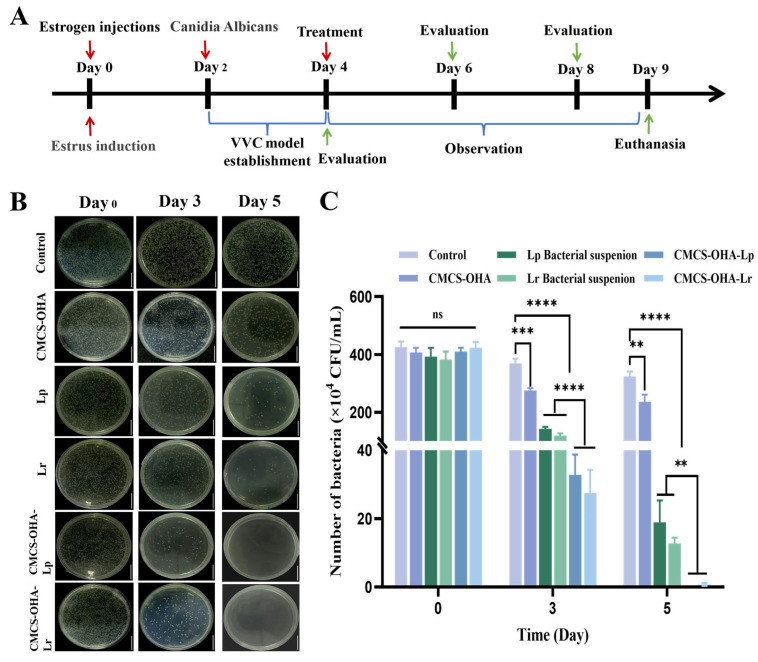
Analysis of the antifungal effect of CMCS-OHA-Lp and CMCS-OHA-Lr hydrogels in vivo with control (model group). (**A**) Flowchart of animal experiments. (**B**) Images depicting the proliferation of *C. albicans* on an agar plate from the vaginal secretions of rats in each group at days 0, 3, and 5 post-administration (scale bar: 1 cm). (**C**) Quantitative analysis of colony-forming units (CFU) (*n* = 6). The results represent the mean ± SD. (ns *p* > 0.05, ** *p* < 0.01, *** *p* < 0.001, and **** *p* < 0.0001).

The timeline for the establishment of the VVC model, drug administration, and sample collection in experimental animals is illustrated in Figure 8A. Rat vaginal secretions were collected daily to assess the estrus cycle (Figure S2). The pre-estrus stage was characterized by the predominance of nucleated epithelial cells, while during estrus, keratinized non-nucleated epithelial cells were dominant. As shown in Figure S2, *C. albicans* proliferated on the ager plate, indicating successful colonization of the vaginas of SD rats in each group. Additionally, the growth curve was constructed by measuring the optical density (OD). The initial OD_600_ of *C. albicans* was approximately 0.2, and all samples multiplied, reaching a stable phase within 24 h. The OD value reached approximately 1 at the 24 h mark, indicating that the vaginal secretions of SD rats in each group contained a substantial quantity of *C. albicans*. In conclusion, *C. albicans* effectively colonized and infected the vaginal mucosa of SD rats.

### 3.9. In Vivo Antifungal Properties, Inflammatory Changes, and Immunohistochemical Analysis

These samples were cultured on agar plates to observe colony formation and perform a quantitative analysis (Figure 8B,C). The CMCS-OHA hydrogel group showed minimal change in colony numbers compared to the control group. By days 3 and 5, the antifungal effects of the CMCS-OHA-Lp and CMCS-OHA-Lr hydrogel were significantly superior to those of the Lp and Lr suspension groups. Notably, by day 5, the agar plates from the CMCS-OHA-Lp and CMCS-OHA-Lr hydrogel groups exhibited almost no *C. albicans* colonies. This enhanced efficacy may be due to the liquid form of Lp and Lr bacterial suspensions, which were less conducive to retention at the inflammation site. The body weight of rats in each group (Figure S3B) decreased during the modeling period, confirming the successful establishment of the vaginitis model. Post-administration, a gradual increase in body weight was observed, particularly in the CMCS-OHA-Lp and CMCS-OHA-Lr hydrogel groups.

**Figure 9:**
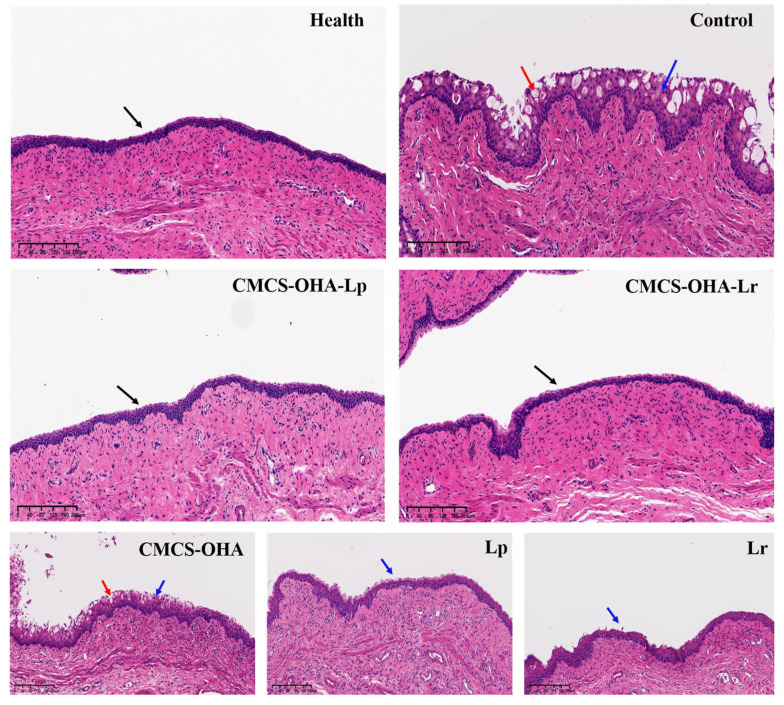
Pathological sections of vaginal tissue from different treatment groups on a rat model of VVC and control (model group). Black arrow: complete and continuous vaginal epithelial tissue; blue arrow: inflammatory cells; red arrow: micro-abscesses and erosion (scale bar: 100 µm).

Histopathological assessment using H&E staining revealed that the healthy group’s vaginal epithelium was smooth and continuous, with no necrosis, exfoliation, or significant inflammatory cell infiltration (Figure 9). In contrast, the control group exhibited pronounced edema, abscess formation, and erosion, characterized by a network structure and substantial inflammatory cell presence. The CMCS-OHA hydrogel group resembled the control group, showing no significant differences. The Lp and Lr bacterial suspension groups showed slight improvements, with reduced edema and some erosion improvement, yet notable inflammatory cell infiltration persisted. The CMCS-OHA-Lp and CMCS-OHA-Lr hydrogel groups demonstrated marked improvements, effectively repairing vaginal tissue. There were no signs of necrosis or tissue loss, erosion had disappeared, and inflammatory cell infiltration was significantly reduced, nearly returning to a healthy state.

**Figure 10:**
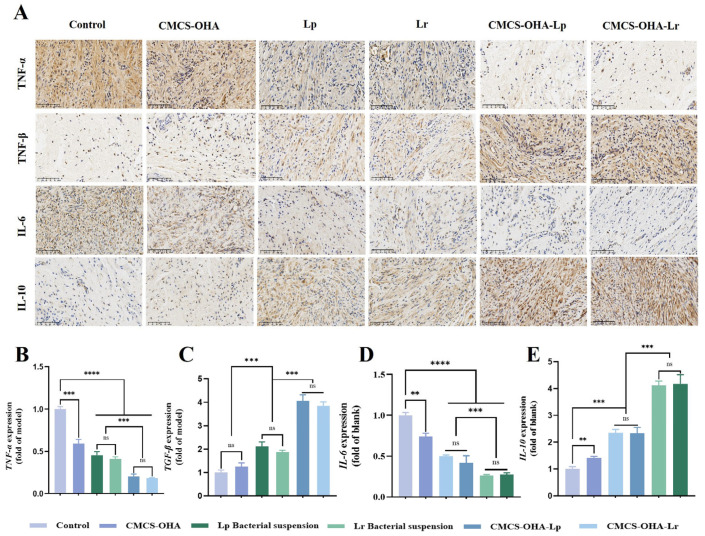
Analysis of the therapeutic effects of CMCS-OHA-Lp and CMCS-OHA-Lr hydrogels on a rat model of vulvovaginal candidiasis (VVC) and control (model group). (**A**) Immunohistochemical staining images of TNF-α, TGF-β, IL-6, and IL-10 in various groups of vaginal tissues (scale: 100 µm). (**B**) Relative gene expression levels of TNF-α, (**C**) TGF-β, (**D**) IL-6, and (**E**) IL-10 across different groups (*n* = 6). The results represent the mean ± SD. (ns *p* > 0.05, ** *p* < 0.01, *** *p* < 0.001, and **** *p* < 0.0001).

The inflammatory responses, which hinders the regeneration and proliferation of vaginal epithelial structures, is characterized by pro-inflammatory cytokines such as TNF-α (Figure 10B) and IL-6 (Figure 10D). As shown in Figure 10A, the control group exhibited elevated pro-inflammatory factors, indicating an excess of inflammatory cells. Treatment with CMCS-OHA-Lp and CMCS-OHA-Lr hydrogels significantly downregulated these cytokines. Additionally, these hydrogel groups exhibited higher levels of anti-inflammatory cytokines, such as TGF-β (Figure 10C) and IL-10 (Figure 10E), compared to the control and other bacterial suspension groups. These findings indicated that CMCS-OHA-Lp and CMCS-OHA-Lr hydrogels exhibited a notable capacity to inhibit the proliferation of *C. albicans* and have the potential of reducing inflammation, promoting the transition to the proliferation stage, thereby facilitating the comprehensive resolution of VVC.

### 3.10. In Vivo Biocompatibility of CMCS-OHA-Lp and CMCS-OHA-Lr Hydrogels

**Figure 11:**
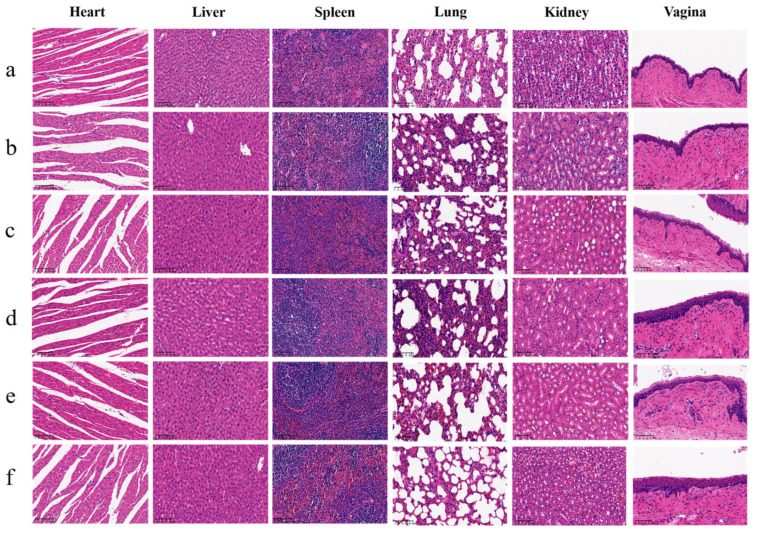
Representative images of H&E-stained sections. (**a**): Untreated; (**b**): CMCS-OHA; (**c**): Lp bacterial suspension; (**d**): Lr bacterial suspension; (**e**): CMCS-OHA-Lp; (**f**): CMCS-OHA-Lr; (scale bar: 100 µm).

As shown in Figure 11, after five days of administration, the tissue structures of the heart, liver, spleen, lungs, kidneys, and vagina in each group remained intact, with no significant lesions observed. Furthermore, the body weight of the SD rats was recorded daily during the administration period, and a graph depicting the changes in body weight was created (Figure S3A). The body weights of rats across all groups exhibited a consistent upward trend, indicating that the rats maintained good health. In conclusion, the findings indicated that CMCS-OHA, CMCS-OHA-Lp, CMCS-OHA-Lr hydrogel, and Lp and Lr bacterial suspensions did not produce significant toxic side effects and were considered safe for in vivo use.

## 4. Discussion

Vaginitis, a common female health problem, is caused by the imbalance of the vaginal microecosystem and the over reproduction of pathogenic microorganisms. Vulvovaginal candidiasis (VVC) is a vaginal inflammation caused by Candida albicans invading the vaginal mucosa, and ranks with bacterial vaginitis as the most common type of vaginal inflammation [1,2,3]. Traditional antibiotic treatments often result in microbial resistance and disruption of the vaginal microenvironment, leading to persistent issues for the patient [9,14]. Addressing these challenges necessitates the exploration of novel therapeutic strategies. Recent advancements in the understanding of the vaginal microenvironment have highlighted the potential of Lactobacillus species, active microorganisms, as promising agents for treating female vaginal diseases [26,58,59]. However, the high sensitivity of probiotics to environmental changes results in low survival rates during processing and storage [36]. Therefore, selecting an appropriate delivery carrier to enhance the viability and bioavailability of probiotics is crucial for achieving their physiological functions.

In order to solve the problem of vaginal microenvironment destruction and drug resistance caused by traditional treatment with antibiotics [15,16,18], this study introduced a new antifungal strategy for vaginitis by developing a microenvironment-responsive injectable vaginal hydrogel loaded with Lactobacillus to regulate the vaginal microecological environment to combat Candida albicans infection. On the one hand, hydrogel encapsulation established a strong protective barrier for Lactobacillus and improved the storage activity. On the other hand, the pH-responsive release of Lactobacillus in the acidic vaginal environment was realized, so as to inhibit the proliferation of Candida albicans and relieve vaginal inflammation.

Firstly, CMCS and OHA were synthesized by modifying the natural hydrogels CS and HA, and the successful oxidation of HA and the effective cross-linking of CMCS-OHA hydrogels were verified by FT-IR and ^1^H NMR [49]. The rheological properties, injectability, and in vitro degradation characteristics of CMCS-OHA were analyzed, with the optimal formulation determined when the volume ratio of CMCS to OHA was 1:1 and the concentration of OHA was 5%. CMCS-OHA-Lp and CMCS-OHA-Lr hydrogels containing Lp and Lr were developed on the basis of CMCS-OHA hydrogels. Degradation, microstructure, pore size, and cytotoxicity were comprehensively evaluated. The results showed that the CMCS-OHA-Lp/Lr hydrogel had a stable structure, uniform pore size, good biocompatibility, and excellent pH-dependent degradation ability. The cumulative release rates of Lp and Lr were 83.50 ± 2.70% and 73.31 ± 2.22% in pH 5.5 medium, respectively, proving the pH-responsive release characteristics of the hydrogel system. The storage activity of Lactobacillus indicated that the survival rates of free Lp and Lr were 70.53 ± 0.91% and 68.17 ± 0.25%, respectively, after 30 days of storage at 4 °C. The survival rates of CMCS-OHA-Lp and CMCS-OHA-Lr hydrogel were 86.90 ± 0.20% and 85.50 ± 0.56%, respectively, proving that hydrogel encapsulation was beneficial for preserving Lactobacillus storage activity.

Plate counting results showed that the antifungal rate of the CMCS-OHA-Lp and CMCS-OHA-Lr hydrogel groups exceeded 80%, and the corresponding antifungal zone diameter was significantly larger than that of the CMCS-OHA hydrogel group, indicating that the loading of Lp and Lr significantly enhanced the antifungal effect of the CMCS-OHA hydrogel. After the co-incubation of CMCS-OHA-Lp 10^8^ (CFU) and CMCS-OHA-Lr 10^8^ (CFU) with *Candida albicans* biofilm, the OD_562_ value decreased significantly to 0.60 ± 0.14 and 0.58 ± 0.12, respectively, proving that the hydrogel system also had a good inhibitory effect on mature biofilms. The in vivo pharmacodynamics results further confirmed the excellent antifungal effects of the CMCS-OHA-Lp and CMCS-OHA-Lr hydrogels. The H&E section showed that the hydrogel system could effectively reduce inflammatory cell infiltration and repair vaginal tissue. Immunohistochemical analysis verified that the hydrogel system could effectively reduce the production of pro-inflammatory cytokines TNF-α and IL-6 and enhance the expression of anti-inflammatory cytokines TGF-β and IL-10, thereby achieving the effect of alleviating inflammation.

However, this study still has several limitations. Firstly, the mechanism of action of CMCS-OHA-Lp and CMCS-OHA-Lr hydrogels in the treatment of VVC still needs to be further studied to lay a foundation for the precise treatment of vaginitis. Secondly, whether the delivery system has good ability to organize detention needs further investigation.

## 5. Conclusions

In this study, a multifunctional shear-thinning hydrogel system, based on a dynamic cross-linked Schiff base network, was successfully developed as an effective probiotic delivery vector for treating VVC. This CMCS-OHA delivery system demonstrated excellent pH responsiveness and self-healing capabilities, which are crucial for its function. The system enables the flexible control and release of *L. plantarum* (Lp) and *L. rhamnosus* (Lr), both of which exhibited remarkable antifungal properties. Importantly, the CMCS-OHA hydrogel significantly enhanced probiotic stability under varying environmental conditions, thereby extending shelf life, preventing inactivation, and improving bioavailability. Both the CMCS-OHA-Lp and CMCS-OHA-Lr hydrogels were non-toxic and exhibited good cytocompatibility with VK2 E6E7 cells. Furthermore, in vivo experiments revealed that these hydrogels effectively inhibited the proliferation of *C. albicans* in the vaginal tissue of rats, promoted the expression of TGF-β and IL-10 protein factors, reduced inflammation, and facilitated the regeneration and proliferation of vaginal epithelial tissue. With these characteristics, this newly developed hydrogel offers a comprehensive strategy for VVC treatment and effectively modulates the therapeutic process. Findings from both in vitro and in vivo studies suggest that CMCS-OHA-Lp and CMCS-OHA-Lr hydrogels hold significant promise for VVC treatment and possess substantial clinical translational potential.

## Data Availability

Data will be made available upon request.

## Supplementary Materials

The following supporting information can be downloaded at: https://www.mdpi.com/article/10.3390/pharmaceutics17040527/s1, Figure S1: Characterization of CMCS-OHA hydrogel; Figure S2: The shear-thinning and self-healing properties of the CMCS-OHA hydrogel were characterized; Figure S3: Successful construction and characterization of the rat VVC model; Figure S4: Changes in body weight of SD rats in each group.
