# Supplementary material for: A Fungistatic Strategy Using a Shear-Thinning pH-Responsive CMCS-OHA-Lp/Lr Hydrogel for Vulvovaginal Candidiasis
Source: Pharmaceutics. 2025 Apr 17;17(4):527. doi: 10.3390/pharmaceutics17040527 (PMC12030408; doi:10.3390/pharmaceutics17040527)
Supplement: Supplementary file 1 [file pharmaceutics-17-00527-s001.zip › pharmaceutics-3564262-supplementary.pdf]

A Fungistatic Strategy Using an Injectable pH-Responsive CMCS-OHA-Lp/Lr Hydrogel for Vulvovaginal Candidiasis

Yuanmin Zhao [a], Xiu Yang [a], Jiale Han [a], Chaoqi Huang [a], Mengliu Shao [a], Yan Yang [a], Qingliang Yang [a,*], Gensheng Yang [a,*]

[a] College of Pharmaceutical Science, Zhejiang University of Technology, Hangzhou 310014, Zhejiang, PR China.

[*]: To whom correspondence should be addressed: qyang@zjut.edu.cn, yanggs@zjut.edu.cn

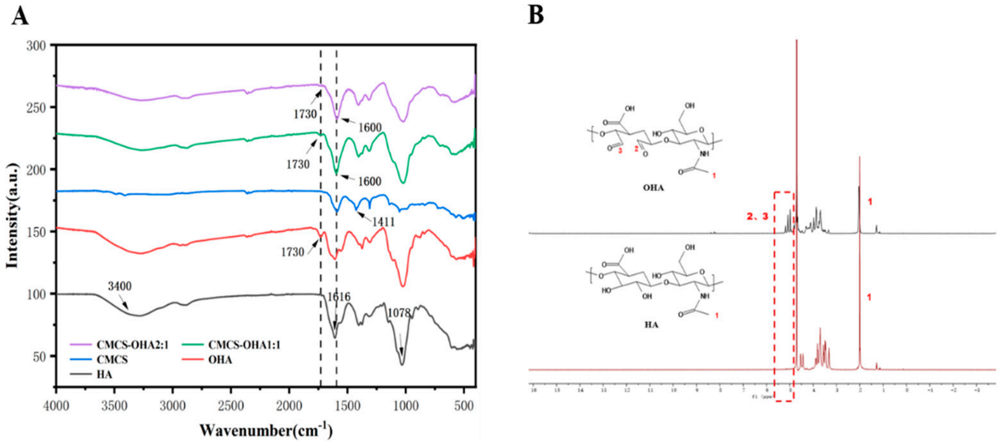

Figure S1. (A) HA, OHA, CMCS, and CMCS-OHA hydrogels FT-IR spectra. (B) HA and OHA ^{1}H-NMR spectra.

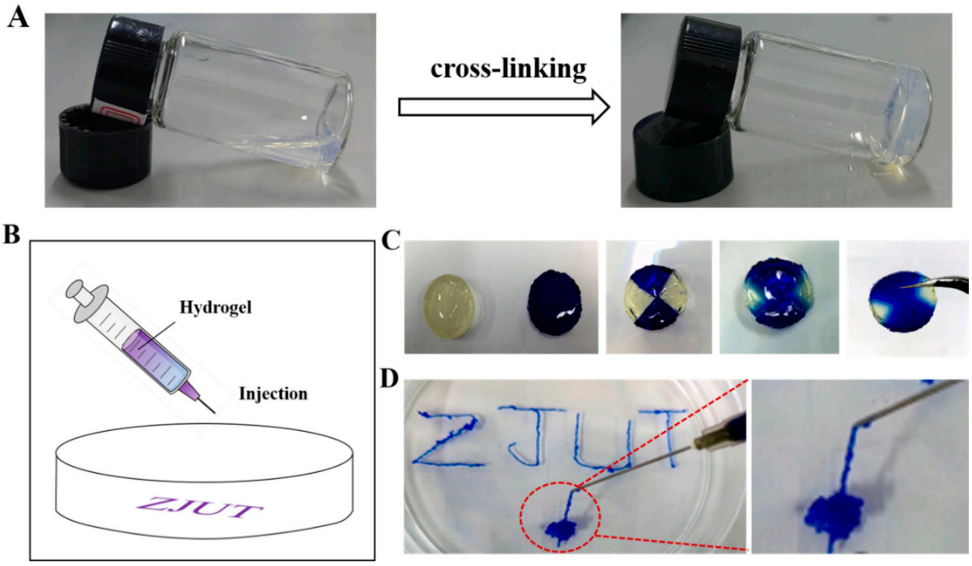

Figure S2. The injectable and self-healing properties of the CMCS-OHA hydrogel were characterized. (A) Sol-gel phase transition of CMCS-OHA (1:1) (B) and (C) Schematic diagrams illustrating injectivity. (C) Diagram depicting self-healing properties

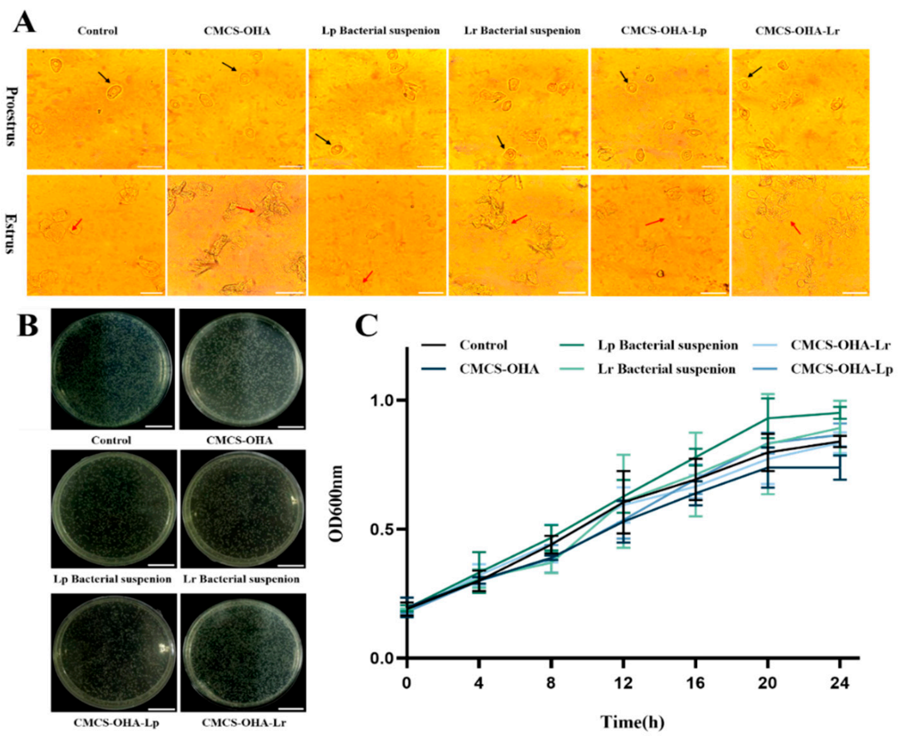

Figure S3. Successful Construction and Characterization of the Rat VVC Model. (A) Morphology of vaginal epithelial cells in Sprague-Dawley (SD) rats during different estrous cycles (scale bar: 20 µm). (B) Proliferation of *Candida albicans* colonies in the vaginal secretions of various groups of SD rats on agar plates (scale bar: 2 cm). (C) Proliferation curves of *Candida albicans* in the vaginal secretions of SD rats across different groups (n=6). The results represent the mean ± SD.

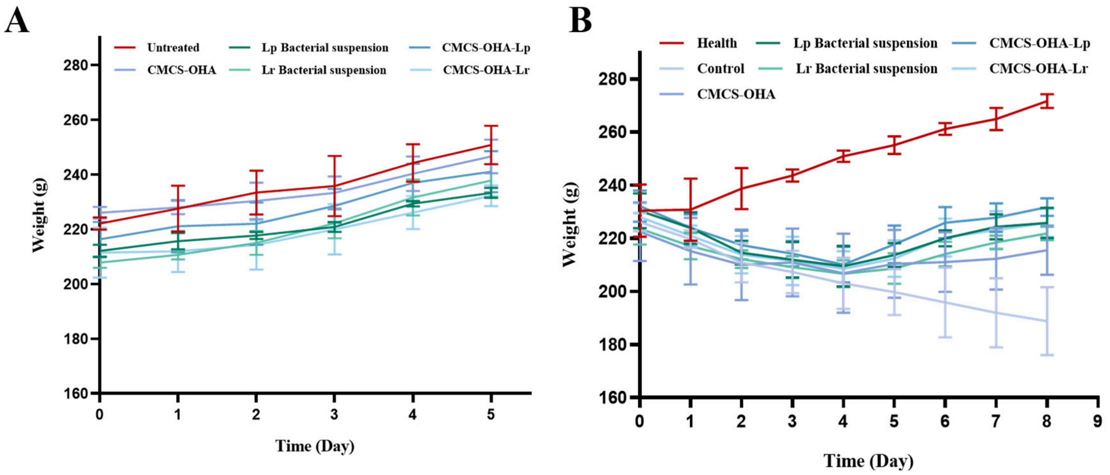

Figure S4. Changes of body weight of SD rats in each group (A) Curves depicting body weight changes of SD rats in each group during the administration period (n=6). (B) Body weight change curve of SD rats in each group throughout the entire experiment (n=6). The results represent the mean ± SD.
